# Who should provide expert opinion in emergency medicine‐related medical litigation?

**DOI:** 10.1111/1742-6723.13962

**Published:** 2022-03-17

**Authors:** Julie Dalling, Anne‐Maree Kelly, Bill Madden, Tina Cockburn

**Affiliations:** ^1^ School of Law Queensland University of Technology Brisbane Queensland Australia; ^2^ Joseph Epstein Centre for Emergency Medicine Research at Western Health Sunshine Hospital Melbourne Victoria Australia; ^3^ Australian Centre for Health Law Research Queensland University of Technology Brisbane Queensland Australia; ^4^ Carroll & O'Dea Lawyers Sydney New South Wales Australia

**Keywords:** expert evidence, litigation, policy

## Abstract

Expert evidence plays a central role in establishing the relevant standard of care in medical litigation. In Australia, little is known about the expert witnesses who provide evidence about the standard of care provided in ED. A sample of recent published case law suggests that a proportion of expert evidence about breach of the standard of reasonable care in ED is provided by medical practitioners who are not emergency physicians and/or have no recent practice experience in an ED. This may potentially distort the identification of the relevant standard of care. In the United States, the American College of Emergency Physicians has attempted to address this issue by developing and promulgating expert witness guidelines. Is there a case for the Australasian College for Emergency Medicine to assume an advocacy role and/or develop standards in this area?

## Introduction

Expert evidence plays a central role in informing legal decision‐makers about the relevant standard of care in medical litigation. Expert evidence about care provided in ED is sought by coroners, the Australian Health Practitioner Regulation Agency or other disciplinary bodies, by solicitors in cases of alleged medical negligence and (less commonly) in criminal cases. By definition, expert witnesses provide opinion evidence, using specialised knowledge arising from their training, study and/or experience.^1^, ^2^ Their role is to inform legal decision‐makers (ultimately judges or juries) on matters outside ordinary experience.^3^ The vast majority of medical expert evidence is provided, at least initially, as written opinions. Oral evidence may never be given in civil claims, because most claims settle prior to trial, but oral evidence is more common in coronial and disciplinary settings.

Expert evidence assists in defining the relevant standard of care in civil liability claims and, where it is considered that the care fails to meet the standard of a reasonable clinician, whether that failure was causative of the outcome.^4^ After considering the expert evidence, and subject to civil liability provisions requiring consideration of widely accepted peer professional practice at the time the care was provided, the Court is the final arbiter of the appropriate standard of care in civil claims, and whether the standard has been met.^4^, ^5^

## Who are the experts that provide opinions on ED standard of care?

We were unable to identify any published information about the characteristics of experts in ED‐related litigation in Australia. Data from other medical litigation contexts suggest that medical expert witnesses in Australia are commonly older, Caucasian, male sex and working in metropolitan and/or academic centres.^6^

There is no publicly accessible record of Australian ED expert witnesses, and the Australasian College for Emergency Medicine (ACEM) does not maintain an expert witness register. However, some information about ED expert witnesses may be gleaned from reviewing published reports of judicial decisions.

## Recent ED‐related case law

The recent Australian cases of *Boxell*,^7^ *Lazareski* ^8^ and *Gould* ^9^ involved care provided in an ED and alleged breach of the standard of reasonable care. In *Boxell*, six of 10 expert witnesses were emergency physicians with current ED practice experience. In *Lazareski*, only one of five experts met this definition. In *Gould*, the number was one in four. The other experts included infectious disease physicians, radiologists, cardiologists and thoracic surgeons. This is not to imply criticism of the experts who were not emergency physicians, who were no doubt providing their opinions in good faith and may have been addressing causation or damages issues rather than standard of care issues. However, in the context of evidence of the standard of care in the ED, a lack of direct and recent experience of the ED context and its challenges could, at least theoretically, result in the identification of a standard that is not consistent with contemporaneous ED practice in Australia.^10^, ^11^ In this context, particular issues identified have been the challenge posed by rare conditions presenting with atypical clinical features, the constraints of ED practice, the balancing of clinical risk, the management of uncertainty, and that it is not feasible in ED to always make a firm diagnosis.

Given this, relevant literature highlights the importance of ‘true’ peers and like circumstances being used to establish the standard of care, and the negative impacts on justice for all parties if inappropriate standards are applied.^6^, ^10^, ^11^

## What can emergency physicians do about this?

### Education and advocacy

There is a clear requirement in the Medical Board of Australia's *Good Medical Practice: A code of conduct for doctors in Australia* for medical practitioners providing evidence to be clear about the limits of their knowledge and expertise and not to provide an opinion beyond those limits.^12^ Similar requirements appear in expert witness codes of conduct published by the courts. For example, an expert is required to state when a particular question, issue or matter falls outside their field of expertise.^13^

One risk may be that non‐emergency physician experts may not appreciate where the extent of their expertise ends with respect to an undifferentiated patient in an ED or the broader ED context. Some may assume that their prior experience working in an ED some years ago equips them with sufficient knowledge of the ED practice context, without appreciating intervening changes in practice or ED‐specific scientific evidence. Another risk may be the application of a diagnosis‐based lens, assuming that because (e.g.) the witness is a cardiologist who treats heart attacks, they may safely comment on assessment of undifferentiated chest pain in ED of which heart disease makes up only a small proportion.

If these risks are accepted, does ACEM have a role in advocacy and education with courts, regulators, insurers and lawyers about the special characteristics of ED practice and the importance of evidence about standard of care being provided by experts with appropriate qualifications and experience? If so, what might be the scope of that role?

### A policy to fill this gap?

The American College for Emergency Medicine (ACEP) first addressed this issue in 1990 in its policy titled *Expert Witness Guidelines for the Specialty of Emergency Medicine*.^14^

Key points in ACEP's guideline are that expert witnesses providing evidence with respect to the appropriate standard of care should:

- Hold current registration as a medical practitioner;
- Have a recognised qualification in Emergency Medicine. (In Australasia, this would be an ACEM Fellowship);
- Be in active clinical practice in Emergency Medicine for at least 3 years (exclusive of training) immediately preceding the events in question; and
- Abide by professional ethical guidelines.

The current version of the ACEP policy also includes a requirement that the expert witness should be willing to submit the transcripts of depositions and testimony to peer review. The policy then goes on to state that misconduct as an expert, including the provision of false, fraudulent, or misleading testimony, may expose the physician to disciplinary action by the College.

This policy has influenced the US legal system. Several US states now have local requirements (either in State statutes or evidence codes) that specify evidence about the applicable standard of care is limited to the standard of care for the specific specialty of the defendant provider.^15^ In the context of a negligence claim against an emergency physician, this means that the defendant is to be held to the standard of care of an average qualified physician practicing in the area of emergency medicine.

Arguably there is some equivalence in Australia in the peer professional practice provisions contained in civil liability legislation. For example, the NSW legislation states that a professional does not incur a liability in negligence arising from the provision of a professional service if it is established that the professional acted in a manner that (at the time the service was provided) was widely accepted in Australia by peer professional opinion as competent professional practice.^5^ Other jurisdictions have similar provisions. However, these provisions do not preclude non‐emergency physicians providing opinions on ED standard of care. It is for the legal representatives in the particular case to persuade the Court as to the probative value (or otherwise) of the opinions put forward by various expert witnesses in a case.

It should be recognised that in the United States, some courts have found similar policies to cast a shadow on the standing of the professional College. For example, one judgement stated that Colleges had, through their guidelines, ‘moved away from disinterested scientific inquiry and into litigation policy to serve their members interests’.^16^

If ACEM was to develop a policy, the question arises whether it should go further than recommending basic qualifications and ethical standards with respect to providing expert evidence. As a professional body with a focus on quality and safety for the benefit of society, these altruistic values should also be applied to the provision of expert evidence by emergency physicians. Ways of achieving this might include recommending that emergency physicians providing expert opinions have training in legal systems and undertake relevant continuing professional education. However this goes beyond the remit of a specialist medical College, whose purpose is education and training, to accredit experts. In any event, an exclusionary accreditation process may not be accepted by the Courts.

## Conclusion

Available evidence suggests that expert opinion about standard of care in ED is sometimes provided by medical practitioners without specific training or recent experience in emergency medicine. This, at least theoretically, has the potential to distort standard of care assessments by legal decision‐makers. An ACEM policy on expert evidence in medical litigation of ED‐related cases could go some way to addressing this issue.

## References

[1] *Evidence Act 1995* (Cth) s 79.

[2] *Makita (Australia) Pty Ltd v Sprowles* [2001] NSWCA 305.

[3] Kelly AM . Medical experts and evaluations of the standard of care in medical litigation – strengths, weaknesses and potential improvements. J. Law Med. 2019; 27: 455–71. 32129047

[4] Madden B , McIlwraith J , Madden B . Australian Medical Liability, 4th edn. Australia: Lexis Nexis Butterworths, 2021.

[5] *Civil Liability Act* 2002 (NSW) s5O (and jurisdictional equivalents).

[6] Grant G , Studdert D . The injury brokers: an empirical profile of medical expert witnesses in personal injury litigation. Melb. Univ. Law Rev. 2013; 13: 831.

[7] *Boxell & Ors v Peninsula Health* [2019] VSC 830.

[8] *Lazarekski v North Metropolitan Health Service* [2019] WADC 84.

[9] *South Western Sydney Local Health District v Gould* [2018] NSWCA 69; BC201802707.

[10] Stankus JL , Sklar D . The expert witness in emergency medicine. Ann. Emerg. Med. 2014; 63: 731–5. 24486099 10.1016/j.annemergmed.2014.01.002

[11] Heniff M . When is an expert truly an expert? Acad. Emerg. Med. 2016; 23: 1176–7. 27130846 10.1111/acem.12995

[12] Medical Board of Australia's Good medical practice: a code of conduct for doctors in Australia (2020). [Cited 10 Nov 2021.] Available from URL: https://www.medicalboard.gov.au/codes-guidelines-policies/code-of-conduct.aspx

[13] Supreme Court (Chapter 1 Expert Witness Code Amendments) Rule 2016 ((SR NO 52 OF 2016) ‐ REG 6‐ Form 44A (Vic) and jurisdictional equivalents.

[14] American College of Emergency Physicians. Expert Witness Guidelines for the Specialty of Emergency Medicine. [Cited 10 Nov 2021.] Available from URL: https://www.acep.org/globalassets/new‐pdfs/policy‐statements/expert‐witness‐guidelines‐for‐the‐specialty‐of‐emergency‐medicine.pdf 10.1016/s0196-0644(01)70042-x 11574813

[15] State Specific Rules Governing Testifying as an Expert Witness in Medical Malpractice Case. [Cited 10 Nov 2021.] Available from URL: https://www.testifyingtraining.com/state‐specific‐rules‐governing‐testifying‐as‐an‐expert‐witness‐in‐medical‐malpractice‐case/

[16] *Adams v Laboratory Corporation of America* 760 F 3d 1322 (11th Cir, 2014) No 13–10425, 29 July 2014.

